# Effects of Covid-19 on the autonomic nervous system in elite athletes assessed by heart rate variability

**DOI:** 10.1007/s11332-023-01067-7

**Published:** 2023-05-18

**Authors:** Jonas Zacher, Aike Branahl, Hans-Georg Predel, Sylvain Laborde

**Affiliations:** 1 Institute of Cardiology and Sports Medicine, German Sport University Cologne, Cologne, Germany; 2 Institute of Psychology, German Sport University Cologne, Cologne, Germany; 3 Normandie Université, EA 4260, UFR STAPS, Caen, France

**Keywords:** Covid-19, Autonomic nervous system, Athletes, Heart rate variability, Heart rate, Blood pressure

## Abstract

**Introduction:**

Covid-19 is a viral airway and systemic infection which can negatively affect the function of the autonomic nervous system. Cardiovascular autonomic function is essential for peak athletic performance. The aim of this study was to assess the effects of a Covid-19 disease on the autonomic nervous system of German elite athletes using heart rate variability (HRV).

**Methods:**

60 elite athletes (aged 22.88 ± 4.71 years) were recruited, 30 of whom had undergone a Covid-19 disease. Heart rate (HR), blood pressure (BP) and heart rate variability (HRV) were measured during rest and during an orthostatic challenge.

**Results:**

At rest and after orthostatic stress blood pressure and the root mean square of successive differences (RMSDD) were significantly lower in Covid-19 athletes (COV) than in control athletes (CON) (*p* = *0.002* and* p* = *0.004*, respectively); heart rate was significantly higher (*p* = *0.001*). COV showed a significantly greater reduction in blood pressure and elevation of heart rate than CON, but the change in RMSSD did not differ significantly during the orthostatic challenge.

**Conclusion:**

These results show a change in cardiac parasympathetic activity and cardiovascular autonomic function in German elite athletes after Covid-19. These findings further the understanding of effects of the Covid-19 disease on the cardiovascular physiology in athletes. Heart rate variability may be a helpful tool in the return-to-play assessment of elite athletes.

**Supplementary Information:**

The online version contains supplementary material available at 10.1007/s11332-023-01067-7.

## Introduction and background

The *coronavirus disease 2019* (Covid-19), caused by the *severe acute respiratory syndrome coronavirus 2* (SARS-CoV-2), has produced a worldwide pandemic resulting in 7 million reported and 17 million estimated deaths [1]. While initially described primarily as a disease of the respiratory tract often causing pneumonia [2], the often systemic effects of the disease have been well described, including pathology of the blood vessels, heart, kidneys, liver and nervous system [3]. Athletes worldwide have been affected by the pandemic on the one hand by contracting Covid-19 and on the other hand by the many counter-measures to the spread of the disease (i.e., lockdown of training facilities and cancelation of tournaments) [4, 5]. Elite athletes are likely at an elevated risk of contracting Covid-19 due to travel- and training-associated contact patterns [6]. While initial data suggested problematically high numbers of organic complications such as myocarditis among athletes [7, 8], more recent findings from this young and generally immune-competent cohort demonstrate high rates of full recovery without complications [9].

While the host of studies on Covid-19 is vast [2, 3, 10–12], the influence on the autonomic nervous system (ANS) is insufficiently understood. The ANS in its regulatory function of body-homeostasis and cardiovascular activity plays a key role for performance, regeneration and immune function [13]. Autonomic dysfunction may manifest in several ways, including disturbance of thermoregulation, gastrointestinal dysmotility or neurogenic circulatory disturbances (i.e., neurogenic orthostatic hypotension) [14]. Effects of viral infections on the ANS have been studied in other diseases prior to the emergence of Covid-19, such as human-immuno-deficiency virus (HIV) patients [15]. Following initial small trials [15–17], a growing host of evidence depicts autonomous dysregulation in patients during and after a SARS-CoV-2 infection [18].

The systemic inflammation caused by a SARS-CoV-2 infection seems to be the most likely mechanism of affecting the autonomic nervous system [19]. Thus, Covid-19 was shown to disrupt the function of the vagus nerve [20] and patients suffering from long- and post-Covid demonstrated reduced vagally mediated heart rate variability (HRV) (vmHRV) [21]. Using HRV, Kaliyaperumal et al. [22] paradoxically demonstrated a significant increase in cardiac parasympathetic activity in Covid-19 subjects, discussing this as an illustration of autonomic dysfunction. A systematic review includes study results demonstrating increases and decreases of varying HRV parameters during Covid-19 disease, obtained from highly heterogeneous cohorts of patients (ranging from asymptomatic to critically ill) [23]. A HRV reduction in hospitalized patients correlated significantly with disease severity and rate of cardiovascular complications [24] and was also found to be a valid predictor of clinical outcome [25]. Kurtoglu et al. reported a significant HRV reduction 5 months after Covid-19 in fully recovered patients compared to healthy controls [26]. A trial investigating patients after Covid-19 focusing on potential long- and post-Covid outcomes demonstrated HRV differences as markers of ANS dysfunction between patients and controls [27].

Regarding athletic performance, the ANS and its markers are essential to detect training overload and to optimize regeneration phases as well as training intervals and intensities [28, 29]. For athletes at the highest competitive levels, even small changes in performance are highly relevant [30]. One of the most thoroughly studied and most frequently used ANS markers is heart rate variability (HRV). HRV represents the change in the time intervals between successive heartbeats [31, 32]. Among the different HRV parameters that can be calculated in the time-domain, the root mean square of the successive differences (RMSSD) represents the direct parasympathetic influence on the cardiovascular system [33] and describes the ability of the heart to respond to various load-dependent demands [34]. In athletes, this is used preferentially to index vagally mediated HRV (vmHRV) in comparison to frequency-domain HRV parameters such as high-frequency HRV because it is influenced less by respiratory parameters [35]. A healthy system is characterized by a high level of vmHRV, as it is able to adapt quickly and variably to different stressors [36]. Patients with autonomic dysfunction or reduced cardiac regulatory capacity demonstrate significantly lower vmHRV values [37, 38], making vmHRV a valid marker of a diseased regulatory system [39]. The general potential of HRV as a health marker is underlined by the fact that in cardiological patients a reduction in vmHRV is associated with an increased mortality [40].

There is limited data regarding the consequences of COVID-19 on athletes’ physical function [41]. Preliminary results point towards an impairment of the vascular system [42], which may be associated to decreased vmHRV [43]. Furthermore, independently from the consequences of COVID-19, the lockdown situation may have triggered in itself a challenge for the autonomic nervous system, due to reduced training abilities, and a study with healthy German elite handball players has reported a decreased endurance capacity after the lockdown [41].

To the authors’ knowledge, trials regarding the effect of Covid-19 on vmHRV in elite athletes are lacking thus far. As detection of over-training or disease is very important in the day-to-day athlete care and HRV is used by many athletes (primarily in the endurance disciplines) to structure training loads and intensities, understanding the effects of the now very common Covid-19 disease on the ANS in athletes is highly relevant. Thus, the aim of the current trial is to investigate the effect of a prior SARS-CoV-2 infection on the autonomic nervous system in elite athletes using heart rate, blood pressure and HRV and their respective responses to a stressor.

### Hypotheses

Hypothesis I: Athletes after a SARS-CoV-2 infection have a higher resting heart rate (HR), lower resting blood pressure (BP) and a reduced resting vmHRV (root mean square of successive differences, RMSSD) compared to control group athletes.

Hypothesis II: Athletes after a SARS-CoV-2 infection show a reduced autonomic reactivity (vmHRV) to an acute orthostatic stress compared to control group athletes.

Hypothesis III: A reduced autonomic reactivity (vmHRV) correlates with symptom severity during the infection.

## Methods

### Participants

**Fig. 1:**
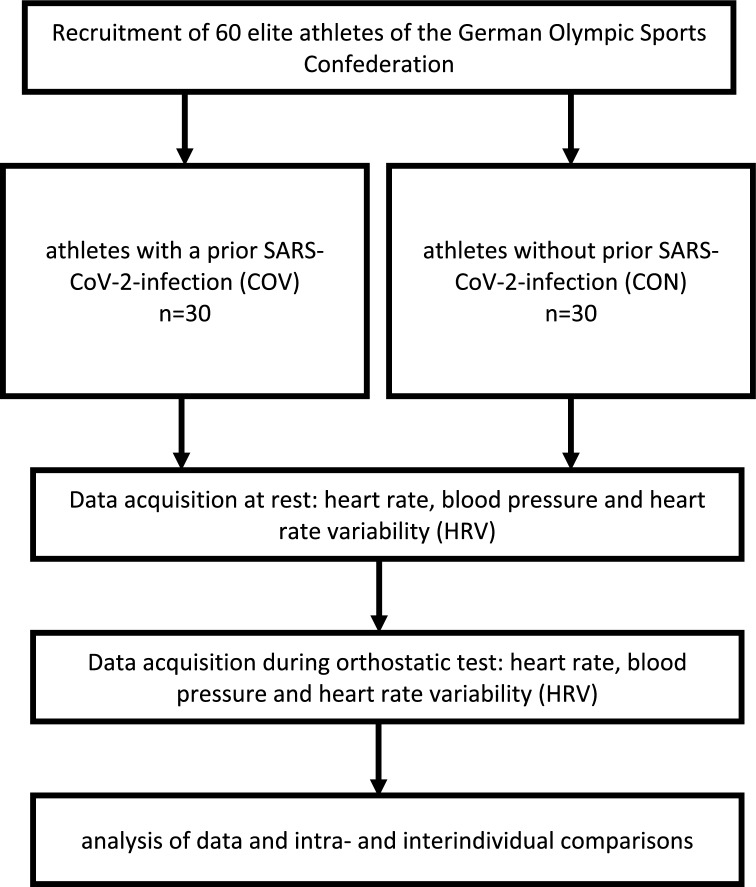
Flowchart of study design

60 elite athletes (for demographic data, see Table 1) of the German Olympic Sports Confederation (*Deutscher Olympischer Sportbund*) were enrolled, of whom 30 had contracted a SARS-CoV-2 infection (*COV*, 12 women and 18 men) and 30 had not (*CON*, 8 women and 22 men). Included were active athletes with written consent who trained > 20 met-h/week from the following disciplines. COV: athletics (7), basketball (2), boxing (1), fencing (5), field hockey (1), gymnastics (2), judo (5), taekwondo (6), and wrestling (1); CON: athletics (3), basketball (3), climbing (2), cycling (2), fencing (2), field hockey (2), golf (2), ice hockey (5), karate (3), rowing (2), swimming (1), table tennis (2), and volleyball (1). Athletes were included into the COV-group if a prior polymerase chain reaction (PCR)-positive infection was documented and consent given. Exclusion criteria: Any prior pathological arrhythmia, cardiovascular diseases or no consent. At the time of testing, all athletes were symptom-free, had echocardiography, electrocardiogram and blood works without relevant pathological findings and had resumed regular training in their respective disciplines. As the trial was conducted before the release of the first Covid-19 vaccines, all athletes were unvaccinated. See Fig. 1 for study design.

**Table 1:** Age, biometric data and training history of Covid-19 group and control group

Variables	Absolute values ± SD		*p* values
	Covid-19 (COV, *n* = 30)	Control (CON, *n* = 30)	COV vs. CON
Age, years	22.77 ± 3.98	23.0 ± 5.4	0.850
Height, m	1.78 ± 0.10	1.80 ± 0.08	0.472
Weight, kg	75.0 ± 16.01	75.83 ± 10.28	0.811
Years of training	13.77 ± 4.73	11.3 ± 5.46	0.051
Hours of training/week	15.00 ± 5.25	16.83 ± 6.22	0.266

Data are expressed as mean ± standard deviation (SD) for Covid-19 (COV) and control group (CON)

### Symptoms

Symptoms were documented via standard medical history questionnaire, including shortness of breath, cough, sore throat, diarrhea, fever, loss of taste or sense of smell and headache. Any medical or other treatment was also documented in this way. To compare levels of disease severity, Covid-19 athletes were grouped into 2 categories based on the recommendations of symptom classification of the German Society of Sports Medicine and Prevention: (1) no or mild symptoms, i.e., subfebrile temperatures, slight feeling of illness, lack of shortness of breath and lack of cardiological symptoms; (2) moderate or severe symptoms, i.e., marked feeling of illness, dyspnea at rest, fever > 38.5 °C, persistent cough > 3 days or hospitalization [37].

### Measurements

All athletes were rested (no exercise within the last 12 h) and in a fasting state for at least 6 h, including freedom of caffeine-containing beverages. Before the commencement of measurements, all athletes rested in a quiet room for 10 min in a comfortable sitting position. During the measurement phase, participants were not required to keep their eyes shut, but to refrain from movement as far as possible. Breathing rates were not specified, participants were instructed to breathe spontaneously.

**Fig. 2:**
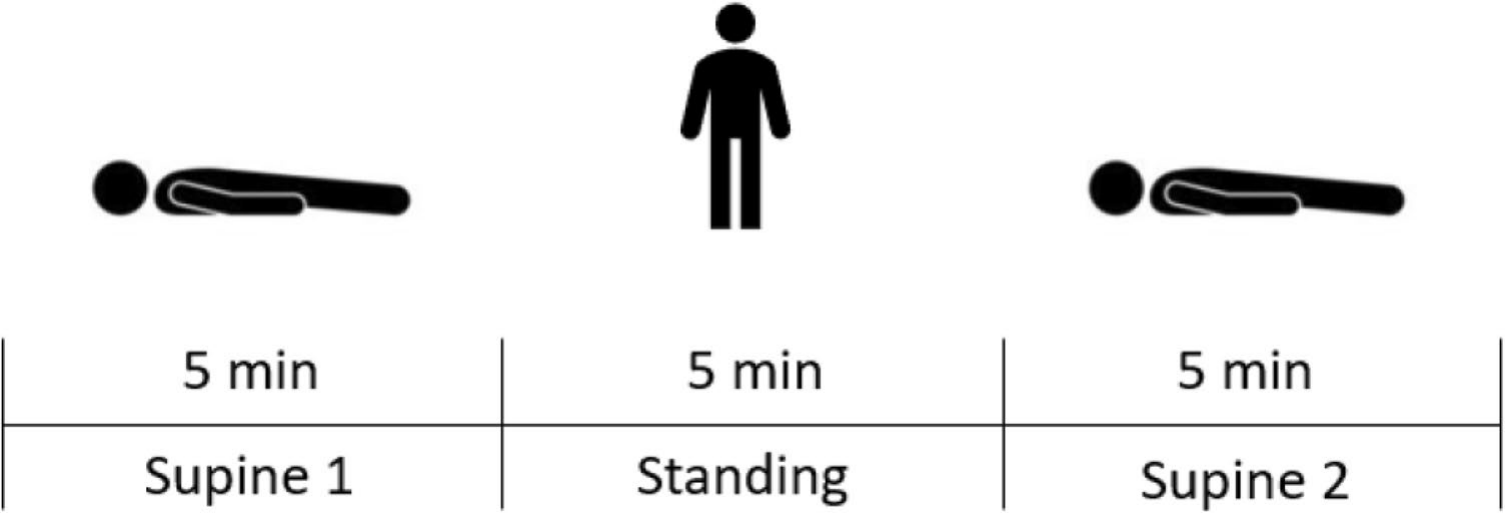
Orthostatic test while HRV parameters are recorded

HRV was measured with an ECG device (Faros 180°, Bittium, Kuopio, Finland) at a sampling rate of 500 Hz. Two disposable ECG pre-gelled electrodes (Ambu L-00-S/25, Ambu GmbH, Bad Nauheim, Germany) were used. The negative electrode was placed in the right infraclavicular fossa (just below the right clavicle), while the positive electrode was placed on the left side of the chest below the pectoral muscle in the left anterior axillary line. From ECG recordings HRV values were extracted with Kubios HRV Software (University of Eastern Finland, Kuopio, Finland). All operations were performed according to the standards of the Task Force of the European Society [44]. All participants performed an orthostatic test (see Fig. 2), while the HRV parameters were recorded: 5 min of supine rest (SU1) were followed by 5 min standing (ST) (acute elevation from supine to standing position), followed by another 5 min of supine rest (SU2). This orthostatic test includes all relevant phases of autonomic function: resting, reactivity and recovery [39, 40]. In addition, it adds additional insights beyond baseline resting measurements given that the organism has to react to a stressor. No evidence has yet been published regarding cardiovascular reactivity to a stress test in individuals with COVID-19, but Hottenrott et al. present an example of the effects of an orthostatic stress test on HRV in an athlete during a viral infection [45].

For the HRV analysis, the first minute of every measuring phase (SU1, ST, SU2) was removed to allow for “physiological stabilization” [44]. RR intervals to obtain HRV were analyzed using Kubios HRV Software. To eliminate artifacts, data were visually inspected beat by beat by an experienced HRV analyst. Following this, HRV values were extracted. Only calculations related to RMSSD as the most reliable parameter to index vmHRV [40] are included in the analysis. For the other HRV parameters, only descriptive statistics are reported as recommended by recent HRV guidelines (see table in supplements). Respiratory frequency was computed via the ECG-derived respiration algorithm of Kubios HRV Software [46]. Parameters of the groups were compared during all phases of the test. In addition, ratios and absolute differences of SU1 and ST and of ST and SU2 were calculated for the RMSSD and heart rate and compared between the groups to analyze the reactivity and recovery process. This method has been used by Hynynen et al. [47] and allows for an assessment of the intra-individual stressor adaptation. To obtain normal distributed values, the RMSSD data were log-transformed before statistical analysis.

Blood pressure values were measured in a variation of the Schellong test [48] on the left upper arm at 4th min, 6th min (directly after standing up), 10th min and 14th min using the automatic blood pressure monitor M500 (Omron, Kyoto, Japan).

The recorded values of RMSSD, heart rate, respiratory rate and blood pressure, as well as the calculated ratios and absolute differences during the orthostatic challenge, were compared between the Covid-19 group (COV) and the control group (CON). In addition, in a subgroup analysis athletes of the Covid-19 group with moderate and severe symptoms (COVS) were compared to the healthy athlete control group.

### Statistical analysis

The data were merged using Excel (Microsoft Corporation, Redmond, USA) and analyzed using SPSS Version 27 (IBM Corporation, Armonk, USA). The normal distribution was calculated using the Kolmogorov–Smirnoff test. Comparisons between groups with normally distributed values were calculated with independent *t*-tests. Comparisons between groups with non-normally distributed values were calculated using the Mann–Whitney-*U*-test. For all tests, a *p*-value < 0.05 was defined as statistically significant. Pre–post-comparisons were calculated with dependent *t*-tests if values were normally distributed or with the Wilcoxon test if values were non-normally distributed.

## Results

The average time from positive SARS-CoV-2-PCR to the HRV measurement was 34.2 ± 12.7 days. Covid-19 group (COV) and control group (CON) did not differ regarding age, height, body weight, years of training or weekly training hours (see Table 1). No included athletes were currently on any relevant medication.

### Heart rate variability

**Fig. 3:**
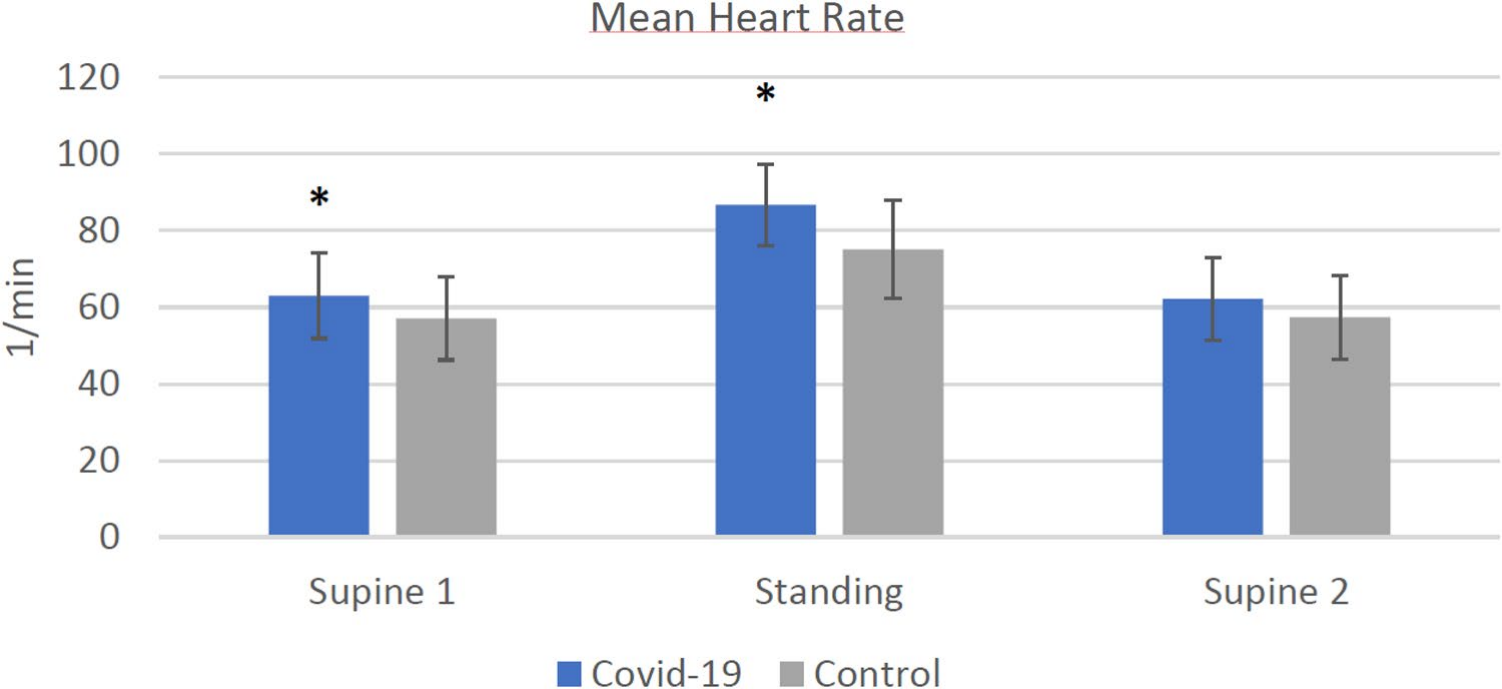
Heart rate of Covid-19 group and control group (* indicates a significant increase compared to the control group)

**Fig. 4:**
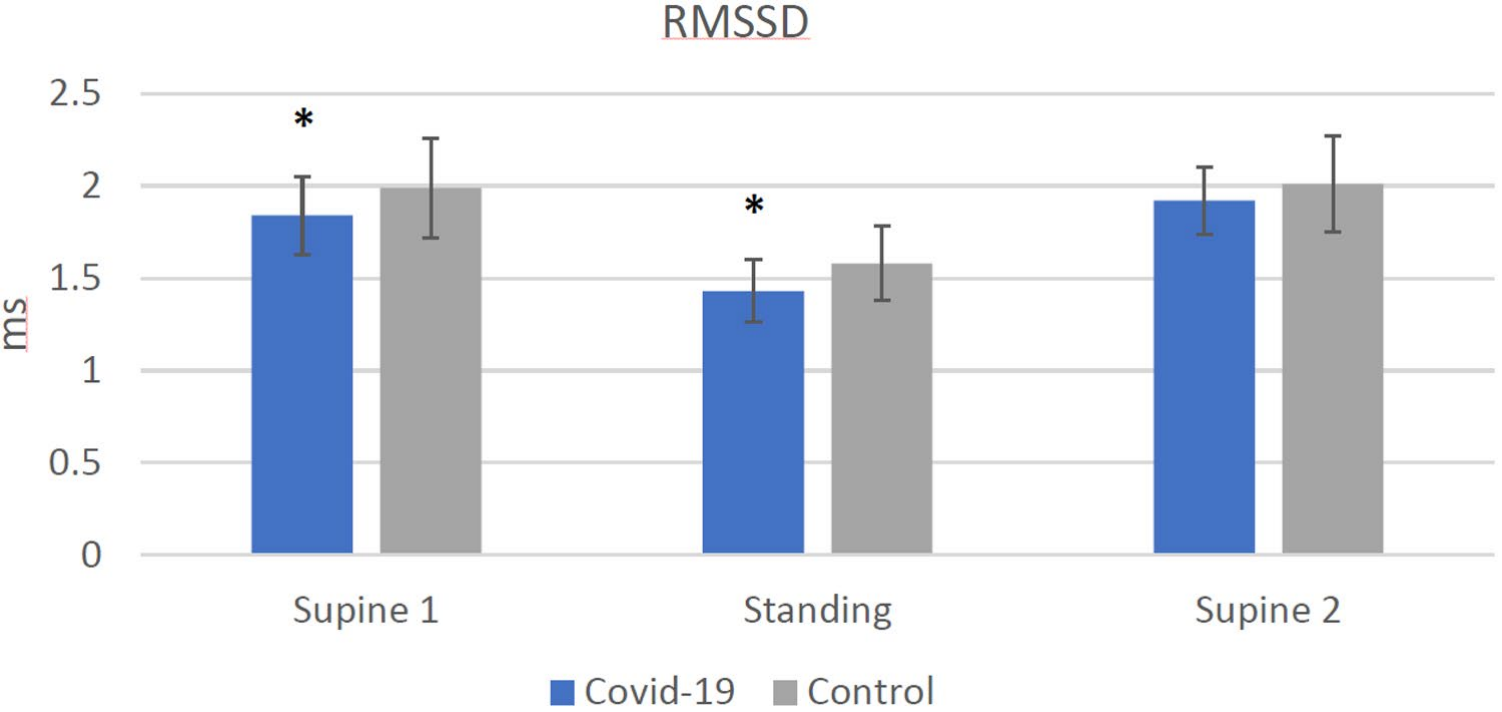
RMSSD value of Covid-19 group and control group (* indicates a significant reduction compared to the control group. RMSSD: Root mean square of successive differences [log-transformed]

Signal quality was high and no measurements had to be excluded due to artifacts or measurement errors. The results of the HRV analysis are summarized in Table 2. Comparisons between groups regarding heart rate and RMSSD are depicted in Figs. 3 and 4, respectively. Resting heart rate was significantly higher in COV than CON during SU1 and ST, while RMSSD was significantly lower. No differences in respiratory rate were detected. After change in position from supine to standing as well as back to supine, the absolute difference in heart rate is significantly higher in COV than CON. The only significant ratio was detected regarding heart rate between ST and SU2 between the groups; the ratios of RMSSD values did not differ.

**Table 2:** Heart rate and heart rate variability

a) Heart rate and heart rate variability of Covid-19 group and control group				
Variables	Absolute values ± SD		*p* values	Effect size
	Covid-19 (COV, *n* = 30)	Control (CON, *n* = 30)	COV vs. CON	COV vs. CON
HR SU1, bpm	62.97 ± 11.1	57.07 ± 10.86	0.042*	0.54
RMSSD SU1, ms	1.84 ± 0.21	1.99 ± 0.27	0.018*	0.63
Respiratory rate SU1, 1/min	11.59 ± 3.7	11.27 ± 3.28	0.725	0.09
HR ST, bpm	86.7 ± 10.74	75.07 ± 12.77	0.001*	0.99
RMSSD ST, ms	1.43 ± 0.17	1.58 ± 0.2	0.004*	0.78
Respiratory rate ST, 1/min	11.41 ± 3.21	11.24 ± 3.04	0.941	0.06
HR SU2, 1/min	62.22 ± 10.71	57.35 ± 10.96	0.087	0.45
RMSSD SU2, ms	1.92 ± 0.18	2.01 ± 0.26	0.143	0.38
Respiratory rate SU2, 1/min	11.27 ± 3.5	11.17 ± 3.14	0.909	0.03
∆ HR SU1-ST	23.74 ± 6.95	18.01 ± 7.56	0.003*	0.79
∆ HR ST-SU2	24.48 ± 6.93	17.72 ± 7.68	0.001*	0.92
∆ RMSSD SU1-ST	0.41 ± 0.2	0.41 ± 0.24	0.924	0.03
∆ RMSSD ST-SU2	0.49 ± 0.21	0.43 ± 0.24	0.321	0.26
HR SU1/ST	0.73 ± 0.077	0.76 ± 0.08	0.082	0.46
HR ST/SU2	1.41 ± 0.15	1.32 ± 0.15	0.029*	0.58
RMSSD SU1/ST	1.3 ± 0.16	1.27 ± 0.18	0.586	0.15
RMSSD ST/SU2	0.75 ± 0.1	0.79 ± 0.1	0.101	0.31

b) Heart rate and heart rate variability of Covid-19 group (moderate and severe symptoms) and control group				
Variables	Absolute values ± SD		*p* values	Effect size
	Covid-19 moderate and severe symptoms (COVS, *n* = 17)	Control (CON, *n* = 30)	COVS vs. CON	COVS vs. CON
HR SU1, bpm	60.39 ± 9.26	57.07 ± 10.86	0.294	0.32
RMSSD SU1, ms	1.85 ± 0.24	1.99 ± 0.27	0.067	0.57
Respiratory rate SU1, 1/min	12.13 ± 4.17	11.27 ± 3.28	0.435	0.24
HR ST, bpm	85.23 ± 9.53	75.07 ± 12.77	0.006*	0.87
RMSSD ST, ms	1.42 ± 0.17	1.58 ± 0.2	0.008*	0.84
Respiratory rate ST, 1/min	11.96 ± 3.77	11.24 ± 3.04	0.479	0.22
HR SU2, 1/min	59.79 ± 9.05	57.35 ± 10.96	0.440	0.24
RMSSD SU2, ms	1.92 ± 0.2	2.01 ± 0.26	0.230	0.37
Respiratory rate SU2, 1/min	11.95 ± 3.81	11.17 ± 3.14	0.455	0.23
∆ HR SU1-ST	24.84 ± 7.47	18.01 ± 7.56	0.004*	0.91
∆ HR ST-SU2	25.44 ± 7.13	17.72 ± 7.68	0.001*	1.03
∆ RMSSD SU1-ST	0.43 ± 0.2	0.41 ± 0.24	0.860	0.05
∆ RMSSD ST-SU2	0.5 ± 0.21	0.43 ± 0.24	0.327	0.3
HR SU1/ST	0.71 ± 0.08	0.76 ± 0.08	0.041	0.64
HR ST/SU2	1.44 ± 0.15	1.32 ± 0.15	0.015*	0.77
RMSSD SU1/ST	1.31 ± 0.15	1.27 ± 0.18	0.490	0.21
RMSSD ST/SU2	0.74 ± 0.1	0.79 ± 0.1	0.125	0.48

Significant differences emphasized in bold. Data are expressed as mean ± standard deviation (SD) for Covid-19 group (COV) with moderate and severe symptoms (COVS) and control group (CON)

*HR* heart rate; *RMSSD* root mean square of successive differences (log-transformed); *∆ HR SU1-ST* absolute difference of the heart rate between supine 1 and standing; *∆ HR ST-SU2* absolute difference of the heart rate between standing and supine 2; *∆ RMSSD SU1-ST* absolute difference of the RMSSD value between supine 1 and standing; *HR SU1/ST* ratio of the heart rate of supine 1 and standing; *HR ST/SU2* ratio of the heart rate of standing and supine 2; RMSSD SU1/ST: ratio of the RMSSD value of supine 1 and standing; RMSSD ST/SU2: ratio of the RMSSD value of standing and supine 1

^*^ ≤ 0.05 significant value

Effect size Cohen’ *d*: ≥ 0.2 small effect; ≥ 0.5 medium effect; ≥ 0.8 large effect

Table 2b shows the comparison between the Covid-19 group with moderate and severe symptoms during the infection (COVS) and the control group. Details regarding the symptoms and their frequency of occurrence are documented in Table 3. Heart rate during the standing phase was found to be significantly higher in COVS, while in the same phase, RMSSD values were significantly lower. Absolute deltas in heart rate upon position change were significantly different between the groups with each position change; this was not found for RMSSD.

**Table 3:** Symptoms and their frequency of occurrence in the Covid-19 cohort

Symptoms	Number of affected athletes (*n* = 30)
No or mild symptoms	13
Moderate or severe symptoms	17
Cough	21
Sore throat	18
Diarrhea	5
Headache	23
Loss of taste or sense of smell	16
Shortness of breath	12
Fever	10

### Blood pressure

Blood pressure values are presented in Table 4 (COV vs. CON). All blood pressure values of the Covid-19 group were significantly lower than those of the control group. In addition, the delta in blood pressure as a reaction to the orthostatic challenge differed significantly between COV and CON: the systolic blood pressure dropped by > 4 mmHg in the COV-group, while remaining stable in the CON-group.

**Table 4:** Blood pressure values of Covid-19 group and control group

Variables	Absolute values ± SD		*P* values	Effect size
	Covid-19 (COV, *n* = 30)	Control (CON, *n* = 30)	COV vs. CON	COV vs. CON
BP4 systolic	117.83 ± 12.27	125.87 ± 10.72	0.006*	0.7
BP6 systolic	113.27 ± 13.13	125.87 ± 12.99	0.002*	0.97
BP10 systolic	112.3 ± 11.75	125.0 ± 9.85	0.000*	1.17
BP14 systolic	117.7 ± 10.97	124.53 ± 10.4	0.016*	0.64
∆ BP4–BP6	4.57 ± 7.96	0.0 ± 8.74	0.039*	0.55
∆ BP 14–BP10	5.4 ± 8.23	– 0.47 ± 6.57	0.003*	0.79
BP4/BP6	1.04 ± 0.07	1.0 ± 0.07	0.033*	0.56
BP14/BP10	1.05 ± 0.08	1.0 ± 0.05	0.003*	0.81

Significant differences emphasized in bold. Data are expressed as mean ± standard deviation (SD) for Covid-19 group (COV) and control group (CON)

*BP* systolic blood pressure value at 4th, 6th, 10th, 14th minute; *∆ BP4*–*BP6* Difference between BP4 and BP6; *∆ BP 14*–*BP10* Difference between BP14 and BP10; *BP4/BP6* Quotient of BP4 and BP6; *BP14/BP10* Quotient of BP14 and BP10

^*^ ≤ 0.05 significant value

Effect size Cohen’ *d*: ≥ 0.2 small effect; ≥ 0.5 medium effect; ≥ 0.8 large effect

## Discussion

In this study, the effects of a SARS-CoV-2 infection on the autonomic nervous system (ANS) of German elite athletes were investigated. To this end heart rate, blood pressure and heart rate variability (HRV) were measured during rest and during an orthostatic test.

Our first hypothesis stated: Athletes after a SARS-CoV-2 infection have a higher resting heart rate (HR), lower resting blood pressure (BP) and a reduced resting vmHRV (root mean square of successive differences, RMSSD) compared to control group athletes. This hypothesis can be accepted regarding all three parameters, which were significant as hypothesized.

Our second hypothesis stated: Athletes after a SARS-CoV-2 infection show a reduced autonomic reactivity (vmHRV) to an acute orthostatic stress compared to control group athletes. This hypothesis has to be rejected, as we did not find significant differences in RMSSD in response to the stressor between the groups. However, blood pressure and heart rate reactions differed between the groups, suggesting that the autonomic reactivity may be affected to a certain degree by a SARS-CoV-2 infection.

Hypothesis III: A reduced autonomic reactivity (vmHRV) correlates with symptom severity during the infection. This hypothesis, too, has to be rejected, as no significant differences in the RMSSD-stressor-response were found.

### Results during rest

We observed an elevated heart rate and lower blood pressure during supine and standing positions in the Covid-19 athletes (COV) compared to the control athletes (CON). These findings are indicative of cardiovascular autonomic dysregulation in the aftermath of a SARS-CoV-2 infection. This also applies to the significantly lower RMSSD values in COV than CON supine and standing up. Prior trials in athletes [42] and non-athletes [18, 23] demonstrated significant differences in resting HR and HRV during and after Covid-19 disease. These changes in autonomic function were documented well beyond the active infection and thus were present in elite athletes recovering from Covid-19 [42] and patients assessed for long- and post-Covid effects [27]. The decrease in vmHRV observed may be associated to vascular impairment, in particular arterial stiffness [43]. Even in asymptomatic patients with no signs of post-Covid significant alterations of HRV were measured in comparison to a control group 5 months after the infection [26]. This finding is in line with our findings that fully recovered athletes with no symptoms still demonstrate heart rate and HRV alterations compared to a healthy athlete control group.

### Results of the orthostatic challenge

To further understand the potential autonomic dysregulation after Covid-19, an orthostatic stress test was implemented in this trial, allowing insights into neurological and cardiovascular function [49]. The orthostatic position change leads to gravity-induced rapid changes of hydrostatic pressure, resulting in compensatory sympathetically regulated adjustment to vasoconstriction and heart rate [50]. This allows an assessment of the functionality of the ANS. The orthostatic test showed significantly greater changes in heart rate and blood pressure in COV than CON, suggesting a regulatory deficit in the Covid-19 athletes compared to the healthy cohort. Sudden exposure to gravity significantly reduced an already lower blood pressure in the COV-group, demonstrating the reduced cardiovascular function of the ANS. These findings are in accordance with trials demonstrating autonomic dysregulation in an orthostatic challenge in post-Covid patients [18, 27].

These effects on the autonomic nervous system during rest and as a reaction to a stressor may be due to infection-related alterations to the cholinergic anti-inflammatory pathway mediated by the vagus nerve [51, 52]. In this context, a hypothalamic over-reaction with consecutive immune-dysregulation and vagal dysfunction could be causal [53]. In addition, the effects of Covid-19 on vascular function and reactivity have to be considered as a causal factor in these results. Endothelial dysfunction resulting in altered vascular responsiveness has been documented in Covid-19 patients during and after the infection [54]. Thus, central effects of the autonomic nervous system may affect vascular response and blood pressure, or, vice versa, vascular dysfunction may mimic or interact with alterations of the autonomic nervous system.

A sex-based effect based on generally lower blood pressure in women is highly unlikely, as the number of female and male athletes was similar in COV and CON; also, if the 4 more female athletes in the COV-group had influenced the significantly lower blood pressure values in this group, it would not explain the significant effect of the orthostatic challenge on blood pressure values in COV. A subgroup-assessment of HRV parameters separated by sex did not yield significant results, but had a low prior probability to do so based on the small sample sizes—regardless of potential differences between the sexes. Similarly, post-infection detraining is most likely not an issue, as recovery times were short and all included athletes were back in full training at the time of data acquisition and had mostly been for weeks. Heterogeneity regarding sporting disciplines was present in both cohorts, therefore most likely not playing a role in the documented differences of ANS regulation.

Why no measurable differences in the effect of the orthostatic stress on the RMSSD values between COV and CON could be documented remains to be elucidated. This may be due to the fact RMSSD indexes specifically the parasympathetic nervous system, while effects on heart rate and blood pressure depict the status of the entire ANS. In addition, while some effects of the Covid-19 disease in the ANS could be found, this young and immuno-competent cohort may be able to adequately compensate the parasympathetic response to the stressor despite the recent infection [55].

Despite their youth and health, 57% of the included COV athletes suffered from moderate or severe symptoms during their Covid-19 disease. However, no association between symptom severity and several markers of the ANS could be demonstrated. Thus, symptom severity does not seem to predict the degree of ANS dysregulation in elite athletes after the infection. This is in accordance with reports that fatigue syndrome following SARS-CoV-2 infection in non-athletes is not associated with symptom severity [56]. However, the subgroup analysis in this trial is limited by its sample size. As Lewek et al. demonstrated HRV to be a predictor of symptom severity in hospitalized patients [57], it could be speculated that in larger athlete cohorts similar observations as well as an association between symptom severity and HRV after an infection may be observed.

### Practical implications

This study furthers the understanding of the effects of SARS-CoV-2 infection on the cardiovascular physiology of elite athletes. Thus, it could be demonstrated that asymptomatic elite athletes after having successfully resumed training still demonstrated a certain modulation of the autonomous nervous system function when compared to healthy controls. On the one hand, these findings may be of practical importance to athletes who already use HRV as a daily tool to monitor their health and plan training intensities and competitions. These athletes could use these findings to better understand and evaluate potential alterations in their HRV measurements after a SARS-CoV-2 infection. On the other hand, our findings may be hypothesis generating for larger follow-up trials assessing autonomic function in athletes by HRV after Covid-19. Once further HRV data from larger cohorts and different time-points after a SARS-CoV-2 infection becomes available, these parameters may be incorporated into updated return-to-play algorithms for athletes after Covid-19 disease.

### Strengths and limitations

To date, this is the first study addressing ANS effects of a SARS-CoV-2 infection in elite athletes. Due to patient selection at one of Germany’s largest sports medicine centers, the cohort is comprised of elite athletes only.

However, the relatively small sample is a limitation. The cross-sectional design is a limitation regarding the high level of interindividual variability of heart rate variability parameters; this could be attenuated by the orthostatic challenge, adding the assessment of an intra-individual effect to the study. In addition, the years of training in elite sports may have influenced our findings, given the average years of training as an elite athlete is higher in the COV-group, only missing statistical significance by a slim margin. Thus, the trend in training years between the groups (*p* = 0.051) hints at potentially more senior athletes being more prone to infections: This may be a consequence of more frequent travels due to higher rates of qualifications for international tournaments; in fact, we did observe a high number of infections taking place in training camps and team travels among Olympic-squad athletes.

## Perspective

Our study for the first time demonstrates autonomic dysfunction measured by HRV in elite athletes after Covid-19. Future studies should aim to include more elite athletes to potentially define HRV-norm-values to help assess whether an athlete has regenerated adequately after Covid-19. In addition, elite athletes infected by Covid-19 may apply techniques aiming to stimulate the parasympathetic nervous system to counterbalance the effects of autonomic dysfunction, for example using slow-paced breathing [57, 58]. Future studies could also include further stressors or stimuli for the ANS such as Valsalva-maneuver, as well as alternative parameters of ANS function. Lastly, future studies should also aim to assess any association between ANS-impairment and performance, i.e., by testing for correlations between HRV and ergometric performance, and include several time-points during and after the infection.

## Supplementary Information

Below is the link to the electronic supplementary material. 

Supplementary file1 (DOCX 15 KB)

## Data Availability

Trial data can be made available upon reasonable request to the corresponding author.
