# Supplementary material for: Effects of Covid-19 on the autonomic nervous system in elite athletes assessed by heart rate variability
Source: Sport Sci Health. 2023 May 18:1–12. Online ahead of print. doi: 10.1007/s11332-023-01067-7 (PMC10191822; doi:10.1007/s11332-023-01067-7)
Supplement: Supplementary file 1 — Supplementary file1 (DOCX 15 KB) [file 11332_2023_1067_MOESM1_ESM.docx]

**Supplementary Table** Selected HRV parameters of Covid-19 group and control group

| Variables | Absolute values ± SD | |  | *p* values | Effect size |
| --- | --- | --- | --- | --- | --- |
|  | Covid-19 (COV, n=30) | Control (CON, n=30) |  | COV vs. CON | COV vs. CON |
| SDNN SU1, ms | 77.33 ± 25.52 | 104.85 ± 42.21 |  | **0.003*** | 0.79 |
| PNN50 SU1, % | 46.34 ± 22.78 | 56.41 ± 22.4 |  | 0.06 | 0.45 |
| LF SU1, ms² | 1600.4 ± 1318.5 | 2954.4 ± 3390.8 |  | **0.033*** | 0.53 |
| HF SU1, ms² | 2584.8 ± 2286.2 | 4691.1 ± 5342.4 |  | 0.117 | 0.51 |
| LF/HF SU1 | 1.22 ± 1.65 | 1.58 ± 0.2 |  | 0.469 | 0.16 |
| SDNN ST, ms | 65.89 ± 15.6 | 82.31 ± 26.73 |  | **0.023*** | 0.75 |
| PNN50 ST, % | 7.11 ± 5.16 | 16.8 ± 12.91 |  | **0.002*** | 0.97 |
| LF ST, ms² | 2214.1 ± 1463 | 2748.1 ± 2462 |  | 0.574 | 0.26 |
| HF ST, ms² | 361.6 ± 374.5 | 607.5 ± 553.2 |  | **0.035*** | 0.52 |
| LF/HF ST | 9.79 ± 8.04 | 6.61 ± 5.46 |  | **0.016*** | 0.46 |
| SDNN SU2, ms | 98.46 ± 27.6 | 120.56 ± 45.15 |  | **0.026*** | 0.59 |
| PNN50 SU2, % | 50.99 ± 19.55 | 56.49 ± 20.82 |  | 0.296 | 0.27 |
| LF SU2, ms² | 1820.9 ± 1840.9 | 3290.3 ± 2882.6 |  | **0.012*** | 0.61 |
| HF SU2, ms² | 3023.1 ± 2664.3 | 4425.8 ± 3796.2 |  | 0.143 | 0.43 |
| LF/HF SU2 | 1.02 ± 1.26 | 0.95 ± 0.58 |  | 0.160 | 0.07 |
| Significant differences emphasized in bold | | |  |  |  |
| Data are expressed as mean ± standard deviation (SD) for Covid-19 group (COV) and control group (CON)  SDNN: Standard deviation of all R–R intervals; PNN50: Percentage of successive normal sinus RR intervals more than 50 ms; LF: Low frequencies; HF: High frequencies; LF/HF: Low frequencies/high-frequencies ratio  *≤ 0.05 significant value  Effect size Cohen’ d: ≥ 0.2 small effect; ≥ 0.5 medium effect; ≥ 0.8 large effect | | | | | |
